# Supplementary material for: Microglial MyD88-dependent signaling influences extracellular matrix development and interneuron maturation in the hippocampus
Source: bioRxiv. 2025 Dec 11:2025.12.08.692987. Preprint. [Version 1] doi: 10.64898/2025.12.08.692987 (PMC12709473; doi:10.64898/2025.12.08.692987)

Supplementary Figure 1

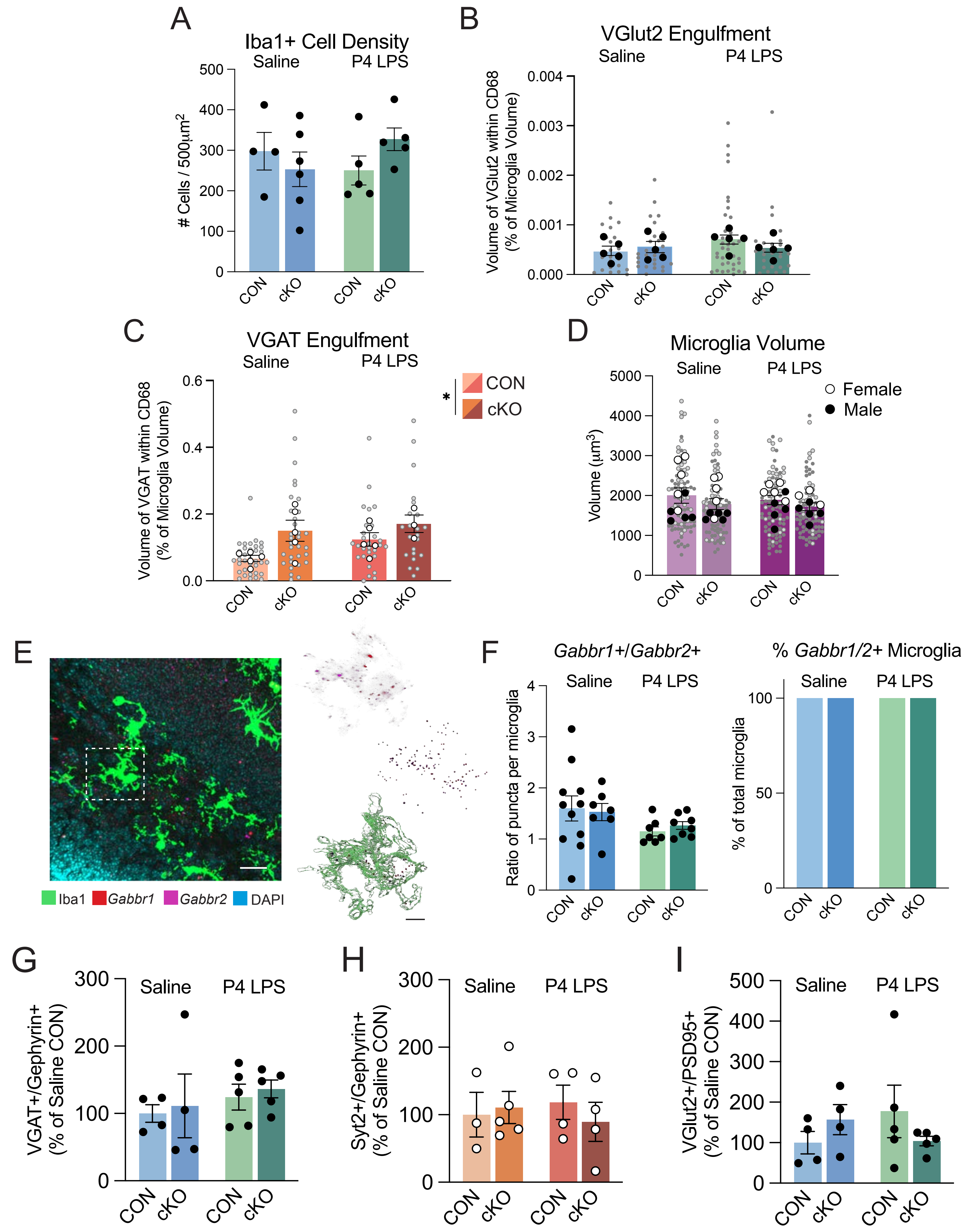

Supplementary Figure 2

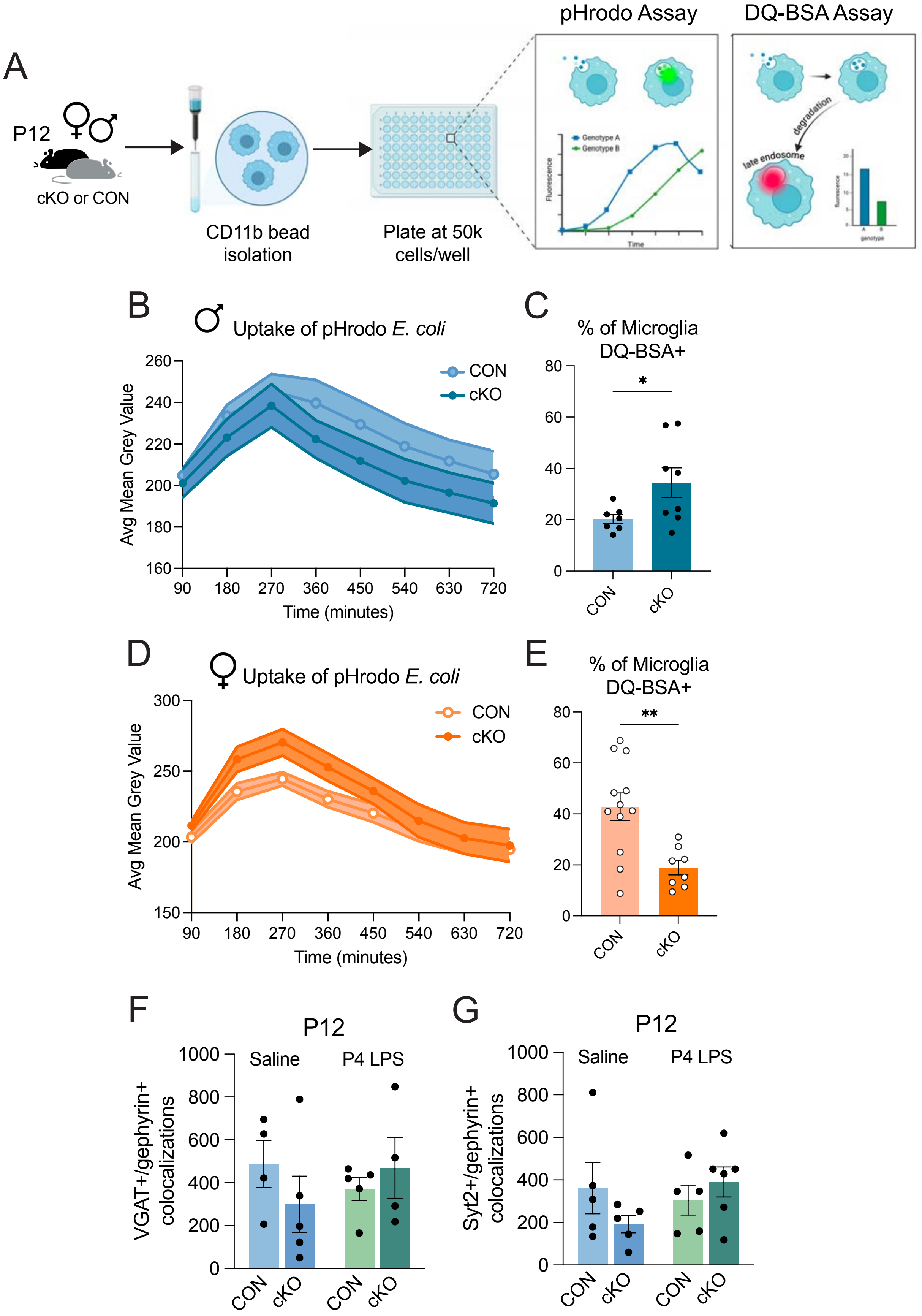

Supplementary Figure 3

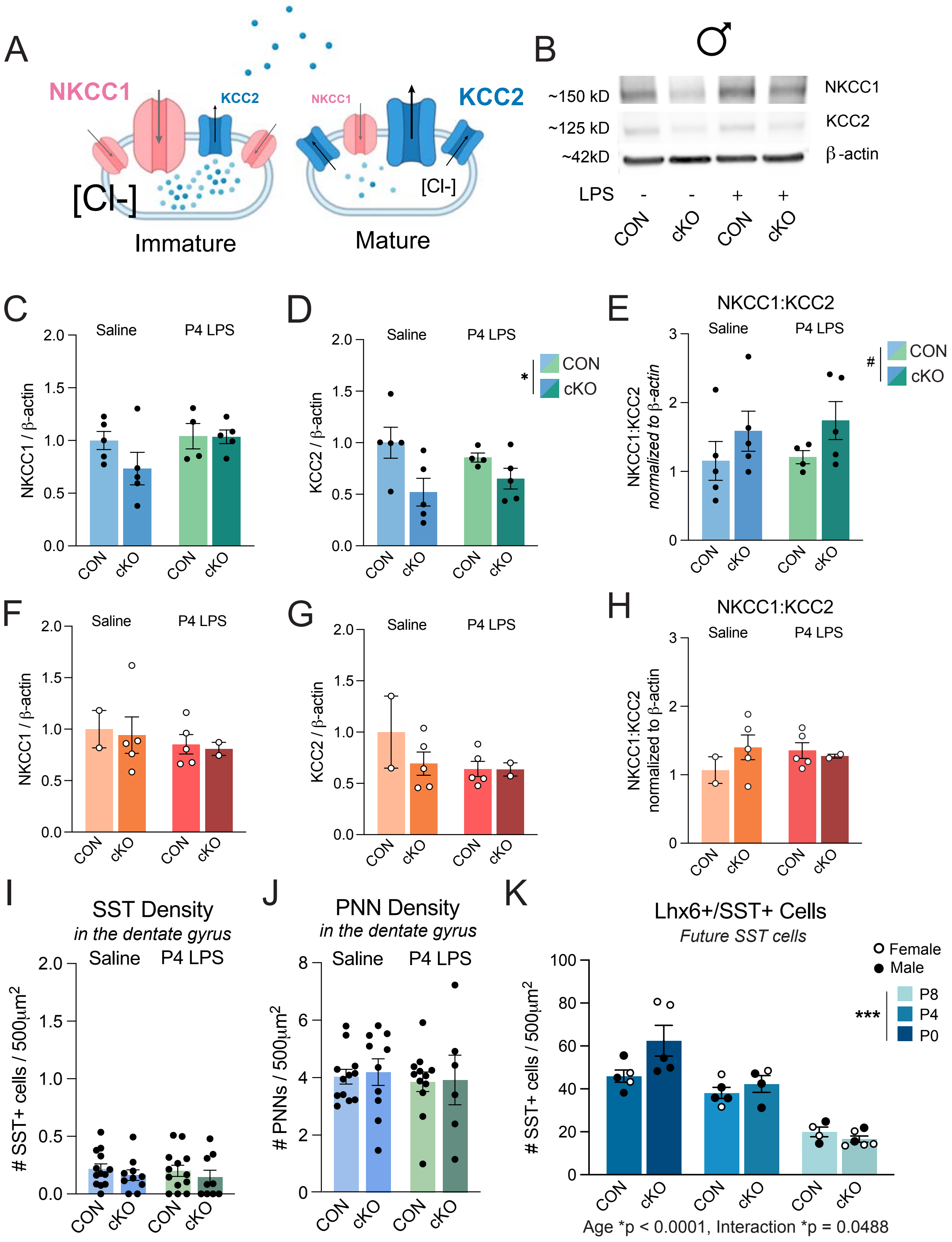

Supplementary Figure 4

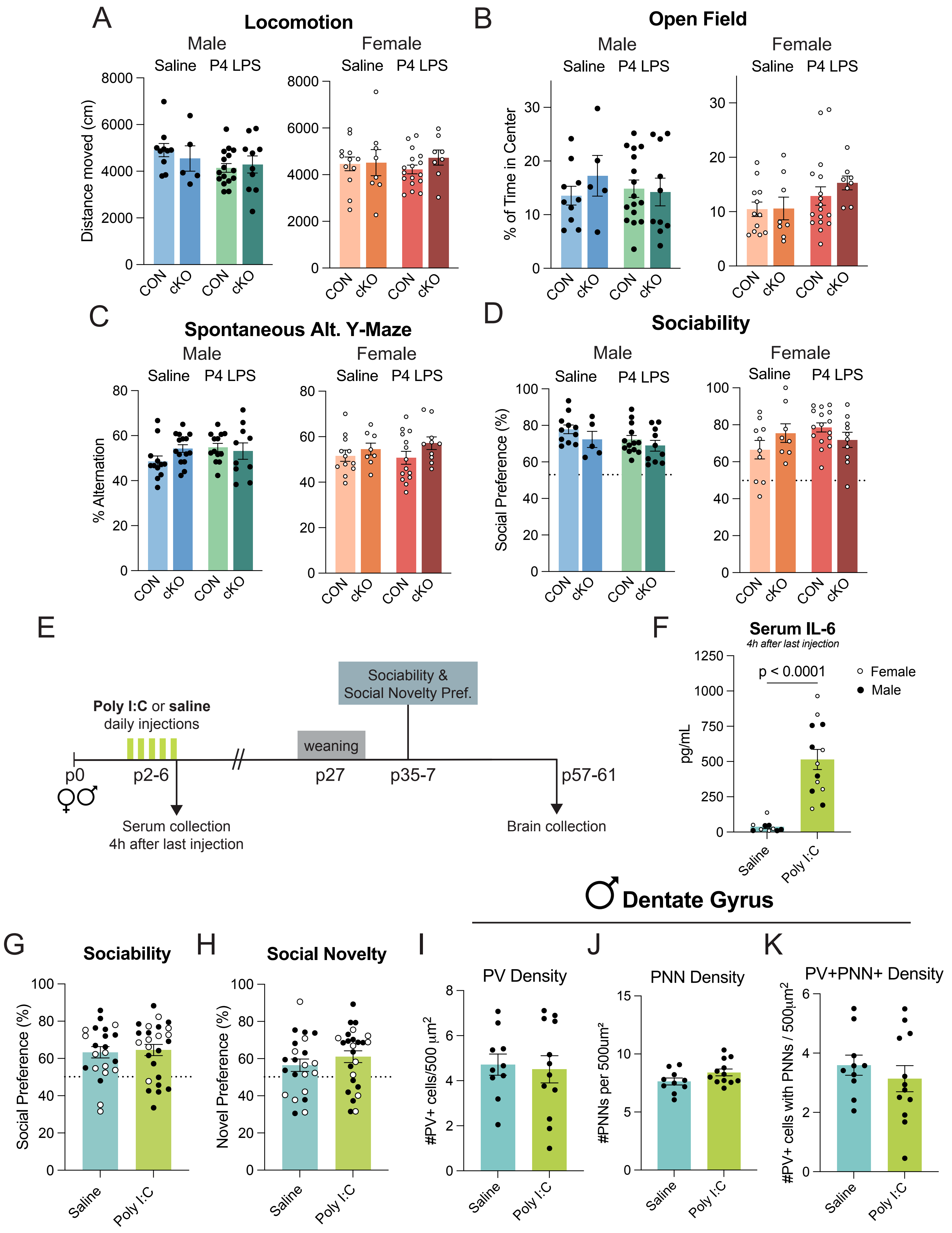

Supplementary Figure 5

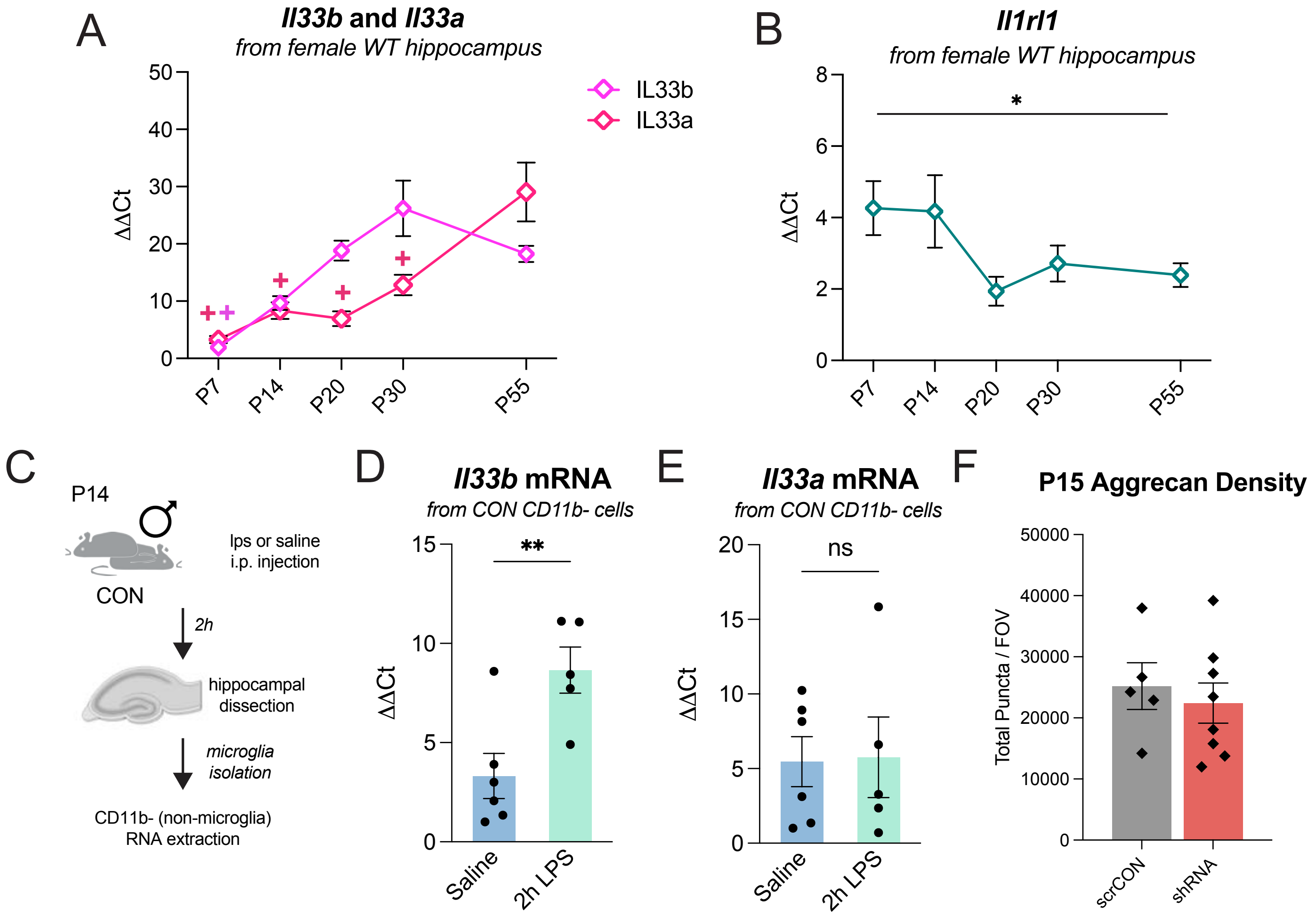

Supplement: Supplement 1 [file media-1.pdf]
