# Supplementary material for: Microglial MyD88-dependent signaling influences extracellular matrix development and interneuron maturation in the hippocampus
Source: bioRxiv. 2025 Dec 11:2025.12.08.692987. Preprint. [Version 1] doi: 10.64898/2025.12.08.692987 (PMC12709473; doi:10.64898/2025.12.08.692987)
Supplement: Supplement 2 [file media-2.pdf]

Supplemental Figure 1. Characterization of male and female early postnatal MyD88-cKO microglia and synapses.

A. Density of male microglia at P12 by Iba1+ somas, normalized to 500μm^2 area. Non-significant by 2-way ANOVA. **B.** Engulfment of excitatory VGlut2 presynaptic protein in male microglia lysosomes at P12, non-significant by 2-way ANOVA. **C.** Engulfment of inhibitory VGAT presynaptic protein within female microglia lysosomes at P12, significant main effect of genotype by 2-way ANOVA. **D.** Microglia 3D volume at P12 in males (closed circles) and females (open circles), non-significant by 2-way ANOVA. **E.** Left, representative image of P12 dentate gyrus, scale bar = 20μm. Right, image of *Gabbr1* (red) and *Gabbr2* (magenta) RNA signal within the inset microglia (top), "Spots" representation of RNA puncta (middle), and representative 3D reconstruction of microglia at P12 with Iba1 (green) protein (bottom), scale bar = 4μm. **F.** Quantification of *Gabbr1* and *Gabbr2* expression in dentate gyrus microglia, as ratio of *Gabbr1*:*Gabbr2* puncta per cell and percent of cells expressing both *Gabbr1* and *Gabbr2*, non-significant by 2-way ANOVA. **G.-I.** P18 quantification of synaptic density in males (**G., I.**) and females (**H.**) in the dentate gyrus. Non-significant by 2-way ANOVA. All error bars = SEM, all statistics run on individual animals (black or black-outlined circles), grey points represent individual microglia analyzed, except for *in situ* experiment (**F.**), where points represent individual microglia, from at least 3 animals/group.

Supplemental Figure 2. P12 MyD88-cKO phagocytosis assays and inhibitory synapse density.

A. Experimental design for phagocytosis assays with microglia isolated from P12 male and female mice. Microglia were plated to 50,000 cells per well, then treated with pHrodo *E. coli* or DQ-BSA and imaged in a live-imaging chamber. **B.** Male pHrodo uptake over time, non-significant by repeated measures 2-way ANOVA. **C.** Male percent of cells expressing DQ-BSA at 270 mins after treatment, significant unpaired t-test. **D.** Female pHrodo uptake over time, non-significant by repeated measures 2-way ANOVA. **E.** Female percent of cells expressing DQ-BSA at 270 mins after treatment, significant unpaired t-test. Each point = 1 animal, average of at least 3 wells/animal. **F.-G.** Inhibitory synapse density quantification in P12 males, non-significant by 2-way ANOVA. All error bars = SEM, all statistics run on individual animals.

Supplemental Figure 3. Additional inhibitory system characterization in the MyD88-cKO hippocampus

A. Schematic of chloride transporter concentrations over development, where increased KCC2 concentration and reduced NKCC1 concentration in maturity leads to normal intracellular chloride concentration, such that GABA signaling hyperpolarizes the cell. A high NKCC1 to KCC2 ratio is therefore considered immature. **B.** Representative western blots from males of NKCC1, KCC2, and β-actin for normalization. **C.** Male abundance of NKCC1, non-significant by 2-way ANOVA. **D.** Male abundance of KCC2, significant main effect of genotype by 2-way ANOVA. **E.** Ratio of male NKCC1:KCC2, trending (p=0.08) main effect of genotype by 2-way ANOVA. **F.-H.** Female quantifications of NKCC1 (**F.**), KCC2 (**G.**), and ratio (**H.**), all non-significant by 2-way ANOVA. **I.** Adult quantification of lowly-abundant somatostatin (SST) expressing interneurons in the male dentate gyrus, non-significant by 2-way ANOVA. **J.** Total number of perineuronal nets (PNNs) in the dentate gyrus of adult males, normalized to 500μm^2 area. Non-significant by 2-way ANOVA. **K.** Quantification of future SST expressing cells by *in situ* hybridization (*Lhx6*-/*Sst*+/DAPI+ cells) in the first postnatal week (P0-P8). Significant main effect of age and interaction (age x genotype) by 2-way ANOVA. All error bars = SEM, all statistics run on individual animals.

Supplemental Figure 4. Additional adult behavioral characterization in MyD88-cKO males and females and early life poly I:C experiment behavior and brain endpoints

A. Total distance moved in open field experiment over 5 minutes in males and females, non-significant by 2-way ANOVA. **B.** Time spent in center of open field apparatus in percent of 5-minute trial. Non-significant in males, trending main effect (p=0.05) of treatment in females by 2-way ANOVA. **C.** Percent correct alternations of total entries in Y-maze working memory paradigm, non-significant by 2-way ANOVA in males and females. **D.** Sociability assay quantified by percent social preference, calculated as time interacting with a social conspecific divided by total time spent interacting with social and non-social stimulus in the 3-chamber assay, *100. Non-significant in males and trending (p=0.05) genotype x treatment interaction in females by 2-way ANOVA. **E.** Experimental design for phenocopy early life poly I:C experiment. Male and female C57BL/6 wild-type mice were injected with poly I:C or saline daily from P2-P6, and social behavior was quantified prior to adult brain collection. Serum was collected in a subset of mice 4h after P6 poly I:C injection to verify immune response. **F.** Serum IL-6 expression by ELISA 4h after last injection, significant by unpaired t-test. **G.-H.** Sociability and social novelty preference, non-significant by unpaired t-test. **I.-K.** Male quantification of PV cell

density (**I.**), PNN density (**J.**), and colabeled PV/PNNs (**K.**), normalized to 500μm^2 area. Non-significant by unpaired t-test.

Supplemental Figure 5. Additional early life IL-33 characterization.

A. Female *Il33b* and *Il33a* isoform expression across wild-type development. Significant main effects of age and isoform x age interaction effect by 2-way ANOVA. + indicates Bonferroni post-hoc significantly different than isoform-matched P55 adult expression. **B.** Female *Il1rl1* expression across wild-type development. Significant by one-way ANOVA, no significant post-hoc differences compared to P55 adult expression, n = 8 mice/age. **C.** Experimental design for non-microglia *Il33b* and *Il33a* expression following peripheral LPS injection. MyD88-CON male mice were injected with saline or 330ug/kg of LPS at P14, then microglia were isolated from hippocampal dissections. CD11b-negative (non-microglia) RNA was extracted and RT-qPCR was performed. **D.** LPS significantly increased *Il33b* expression by unpaired t-test. **E.** Acute peripheral LPS had no significant effect on *Il33a* expression. **F.** Density of total aggrecan puncta from the dentate gyrus (60x magnification) at P15 in mice previously treated with scrambled control virus or shRNA against *Il33* virus at P4, non-significant unpaired t-test. All error bars = SEM, all statistics run on individual animals.
