# Supplementary material for: Microglial MyD88-dependent signaling influences extracellular matrix development and interneuron maturation in the hippocampus
Source: bioRxiv. 2025 Dec 11:2025.12.08.692987. Preprint. [Version 1] doi: 10.64898/2025.12.08.692987 (PMC12709473; doi:10.64898/2025.12.08.692987)
Supplement: Supplement 3 [file NIHPP2025.12.08.692987v1-supplement-3.pdf]

bioRxiv preprint doi: https://doi.org/10.64898/2025.12.08.692987; this version posted December 11, 2025. The copyright holder for this preprint (which was not certified by peer review) is the author/funder, who has granted bioRxiv a license to display the preprint in perpetuity. It is made available under aCC-BY 4.0 International license.

Table S1: Genotyping Primer Sequences

	Forward	Reverse
MyD88	GTTGTGTGTGTCCGACCGT	GTCAGAAACAACCACCACCATGC
Cre recombinase	TTCGGCTATACGTAACAGGG	TCGATGCAACGAGTGATGAG
MyD88 exon 3	TAATGGCAGTCCTCTCCCAG	AGGACTACATTACCCAGGCG

Table S2: Immunohistochemical Antibodies

Antibody	*Source*	*Cat #*	*Concentration*
1° Chicken α Iba1	Synaptic Systems	234009	1:1000
1° Rat α CD68	Biolegend	137002	1:500
1° Guinea Pig α Tmem119	Synaptic Systems	400004	1:500
1° Mouse IgG2a α ZNP-1 (Syt2)	Zirc	ZDB-ATB-081002-25	1:500
1° Mouse IgG1 α gephyrin	Synaptic Systems	147318	1:500
1° Guinea Pig α VGAT	Synaptic Systems	131004	1:1000
1° Guinea Pig α VGlut2	Synaptic Systems	135418	1:2000
1° Rabbit α P2ry12	Anaspec	AS-55043A	1:500
1° Rabbit α Parvalbumin	Swant	PV27a	1:1000
1° Biotin WFA	Vector Laboratories	B-1355	1:1500
1° Rabbit α Aggrecan	Millipore	AB1031	1:500
1° Rabbit α PSD95	ThermoFisher	51-6900	1:500
1° Rabbit α Somatostatin	BMA Biomed (Peninsula)	T-4103	1:500
1° Rabbit α GFP	Synaptic Systems	132002	1:500
2° Chicken 647	ThermoFisher	A-32933	1:300
2° Chicken 488	ThermoFisher	A-21449	1:500
2° Guinea Pig 647	ThermoFisher	A-21450	1:500
2° Guinea Pig 488	ThermoFisher	A-11073	1:500
2° Rat 568	ThermoFisher	A-11077	1:500
2° mouse IgG2a 594	ThermoFisher	A-21131	1:500
2° mouse IgG1 647	ThermoFisher	A-21240	1:300
2° Rabbit 488	ThermoFisher	A-11008	1:500
2° Rabbit 647	ThermoFisher	A-21245	1:500
2° Rabbit 594	ThermoFisher	A-21207	1:500
2° Streptavidin 594	Vector Laboratories	SA-5594-1	1:500

Table S3: Immunoblot Antibodies

Antibody	*Source*	*Cat #*	*Concentration*
1° beta actin	ThermoFisher	MA5-15739	1:2000
1° NKCC1	DSHB Iowa	T4	1:1000
1° KCC2	Cell Signaling	94725	1:1000
1° Syt2	Zirc	ZDB-ATB-081002-25	1:1000
2° Rabbit 647	ThermoFisher	A-21245	1:1000
2° msIgG 488	ThermoFisher	A-11001	1:10,000
2° msIgG1 647	ThermoFisher	A-21240	1:1000
2° msIgG2a 488	ThermoFisher	A-21131	1:1000

bioRxiv preprint doi: https://doi.org/10.64898/2025.12.08.692987; this version posted December 11, 2025. The copyright holder for this preprint (which was not certified by peer review) is the author/funder, who has granted bioRxiv a license to display the preprint in perpetuity. It is made available under aCC-BY 4.0 International license.

Table S4: Custom Probes for RNA-FISH

	RNA TARGET SEQUENCE
Gabbr1	AAAgUUUUUUgUgAggUCUAUAAggAACggCUCUUUgggAAgAAgUAUgUCUggUUUCUCAUCgggUggUAUgCU<br>gACAACUggUUCAAAACCUAUgACCCgUCAAUCAAUUgUACAgUAgAAgAgAUgACUgAggCggUggAgggCCAU<br>AUCACCACggAgAUUgUCAUgCUgAACCCUgCCAACACCCgAAgCAUUUCCAACAUgACAUCACAggAAUUUg<br>UggAgAAACUAACCAAgCggCUgAAAAgACACCCUgAggAgACUggAggCUUCCAggAggCACCACUggCCUAUgA<br>UgCUAUCUgggCCUUggCUUUggCCUUgAACAAgACCUCUggAggAggUggCCgUUCAggAgUgCgCCUggAggAC<br>UUUAACUACAACAACCAgACCAUUACAgACCAAAUCUACCgggCCAUgAACUCCUCCUCCUUUgAgggUgUUU<br>CUggCCACgUggUCUUUgAUgCCAgCggCUCCCggAUggCAUggACgCUUAUCgAgCAgCUACAgggCggCAgCU<br>ACAAgAAgAUCggCUACUACgACAgCACCAAggAUgAUCUUUCCUggUCCAAAACAgACAAgUggAUCggAgggU<br>CUCCCCCAgCCgACCAgACCUUggUCAUCAAgACAUUCCgUUUCCUgUCACAgAAACUCUUUAUCUCCgUCU<br>CAgUUCUCUCCAgCCUgggCAUUgUUCUUgCUgUUgUCUgUCUgUCCUUUAACAUCUACAACUCCCACgUUC<br>gUUAUAUCCAgAACUCCCAgCCCAACCUgAACAAUCUgACUgCUgUgggCUgCUCACUggCACUAgCUgCUgU<br>CUUCCCCCUUgggCUggAUggUUACCACAUAgggAgAAgCCAgUUCCCAUUUgUCUgCCAggCCCgCCUUUggC<br>UCUUgggCUUAggCUUUAgUCUgggCUAUggCUCUAUgUUCACCAAgAUCUggUggg
Gabbr2	CggCgCgUgggCACACUCACgCAggACgUgCAgCgCUUCUCCgAggUgAggAACgACCUgACUggggUUCUgUACg<br>gggAAgACAUUgAgAUCUCAgACACggAgAgCUUCUCCAAUgAUCCCUgCACCAgCgUCAAAAAgCUCAAggggA<br>ACgACgUgCggAUCAUCCUUggCCAgUUUgACCAgAACAUggCAgCgAAAgUCUUCUgUUgUgCCUUUgAggAgA<br>gCAUgUUUggCAgCAAgUACCAgUggAUCAUCCCgggCUggUACgAgCCUgCgUggUgggAACAggUgCACgUggAg<br>gCCAACUCCUCACgCUgCCUgCgCAggAgCCUCCUggCUgCCAUggAAggCUACAUCggAgUggACUUCgAgCCC<br>CUgAgCUCCAAACAAAUCAAgACCAUCUCAgggAAgACUCCACAgCAAUACgAAAgAgAAUACAACAgCAAgCg<br>UUCgggUgUAgggCCCAgCAAgUUCCAUgggUAUgCCUACgAUggCAUCUgggUCAUCgCCAAgACCCUgCAgAg<br>ggCCAUggAgACACUgCAUgCCAgUAgCAggCACCAgCggAUCCAggACUUCAACUACACAgACCACACCCUggg<br>CAgAAUCAUCCUCAACgCCAUgAACgAAACCAACUUCUUCggAgUCACgggUCAAgUUgUgUUCCggAACgggg<br>AAAgAAUgggAACCAUUAAAUUUACUCAAUUUCAAgACAgCAgAgAggUgAAggUCggCgAgUACAACgCUgUgg<br>CUgACACACUggAgAUCAUCAACgACACCAUAAggUUCCAggggUCggAgCCACCCAAggACAAgACCAUCAUU<br>CUAgAgCAgCUUCggAAgAUCUCgCUUCCACUgUAUAgC
